# PERCEIVED AVAILABILITY OF CARE AND SUPPORT AMONG OLDER ADULTS IN BANGLADESH

**DOI:** 10.1093/geroni/igz038.2500

**Published:** 2019-11-08

**Authors:** Farhana Ferdous Luna

**Affiliations:** Syracuse University, Syracuse, New York, United States

## Abstract

Care for older adults is most precarious in developing countries where poverty and weak state support systems have put the well-being of their older populations at risk. Bangladesh is one such nation characterized by excess poverty, poor health, high mortality rates, and illiteracy among its older adults. The lack of elder-friendly infrastructure presents another problem for aging well in Bangladesh. This study examined perceptions about the adequacy of care and support received by older Bangladeshis. A cross-sectional survey collected data from 100 older people who were purposively sampled. Results revealed that older people generally are not satisfied with support services from the government and feel that old-age care has historically declined. Inadequate care and support was cited both at family and state levels. Respondents expressed concern that earlier generations of older people were better taken care of than the present generation, and that the former received more respect than the latter. Factors related to perceived support deficits included poverty, widowhood, and migration of sons. In this patrilineal culture, widowed women in particular perceived themselves as disadvantaged in terms of care availability. We conclude by recommending that policies be designed to enhance care and support services for older people in Bangladesh, particularly the most vulnerable and marginalized among them.

